# Lipid metabolic Reprogramming: Role in Melanoma Progression and Therapeutic Perspectives

**DOI:** 10.3390/cancers12113147

**Published:** 2020-10-27

**Authors:** Laurence Pellerin, Lorry Carrié, Carine Dufau, Laurence Nieto, Bruno Ségui, Thierry Levade, Joëlle Riond, Nathalie Andrieu-Abadie

**Affiliations:** 1 Centre de Recherches en Cancérologie de Toulouse, Equipe Labellisée Fondation ARC, Université Fédérale de Toulouse Midi-Pyrénées, Université Toulouse III Paul-Sabatier, Inserm 1037, 2 avenue Hubert Curien, tgrCS 53717, 31037 Toulouse CEDEX 1, France; laurence.pellerin@inserm.fr (L.P.); lorry.carrie@inserm.fr (L.C.); carine.dufau@inserm.fr (C.D.); bruno.segui@inserm.fr (B.S.); thierry.levade@inserm.fr (T.L.); 2 Institut de Pharmacologie et de Biologie Structurale, CNRS, Université Toulouse III Paul-Sabatier, UMR 5089, 205 Route de Narbonne, 31400 Toulouse, France; laurence.nieto@ipbs.fr; 3 Laboratoire de Biochimie Métabolique, CHU Toulouse, 31059 Toulouse, France

**Keywords:** cancer, cholesterol, eicosanoid, fatty acid, glycerophospholipid, lipid droplet, metastasis, obesity, phenotypic switch, pseudo-EMT, sphingolipid

## Abstract

**Simple Summary:**

Melanoma is a devastating skin cancer characterized by an impressive metabolic plasticity. Melanoma cells are able to adapt to the tumor microenvironment by using a variety of fuels that contribute to tumor growth and progression. In this review, the authors summarize the contribution of the lipid metabolic network in melanoma plasticity and aggressiveness, with a particular attention to specific lipid classes such as glycerophospholipids, sphingolipids, sterols and eicosanoids. They also highlight the role of adipose tissue in tumor progression as well as the potential antitumor role of drugs targeting critical steps of lipid metabolic pathways in the context of melanoma.

**Abstract:**

Metabolic reprogramming contributes to the pathogenesis and heterogeneity of melanoma. It is driven both by oncogenic events and the constraints imposed by a nutrient- and oxygen-scarce microenvironment. Among the most prominent metabolic reprogramming features is an increased rate of lipid synthesis. Lipids serve as a source of energy and form the structural foundation of all membranes, but have also emerged as mediators that not only impact classical oncogenic signaling pathways, but also contribute to melanoma progression. Various alterations in fatty acid metabolism have been reported and can contribute to melanoma cell aggressiveness. Elevated expression of the key lipogenic fatty acid synthase is associated with tumor cell invasion and poor prognosis. Fatty acid uptake from the surrounding microenvironment, fatty acid β-oxidation and storage also appear to play an essential role in tumor cell migration. The aim of this review is (**i**) to focus on the major alterations affecting lipid storage organelles and lipid metabolism. A particular attention has been paid to glycerophospholipids, sphingolipids, sterols and eicosanoids, (**ii**) to discuss how these metabolic dysregulations contribute to the phenotype plasticity of melanoma cells and/or melanoma aggressiveness, and (**iii**) to highlight therapeutic approaches targeting lipid metabolism that could be applicable for melanoma treatment.

## 1. Introduction

The metabolic remodeling is a crucial process that allows melanoma cells to adapt to tumor microenvironment (TME) and to sustain growth and dissemination [1,2]. A comparative metabolic flux profiling of melanoma cell lines and normal melanocytes showed that all melanoma cells consumed more glucose and produced more lactate than melanocytes [3].

**Figure 1:**
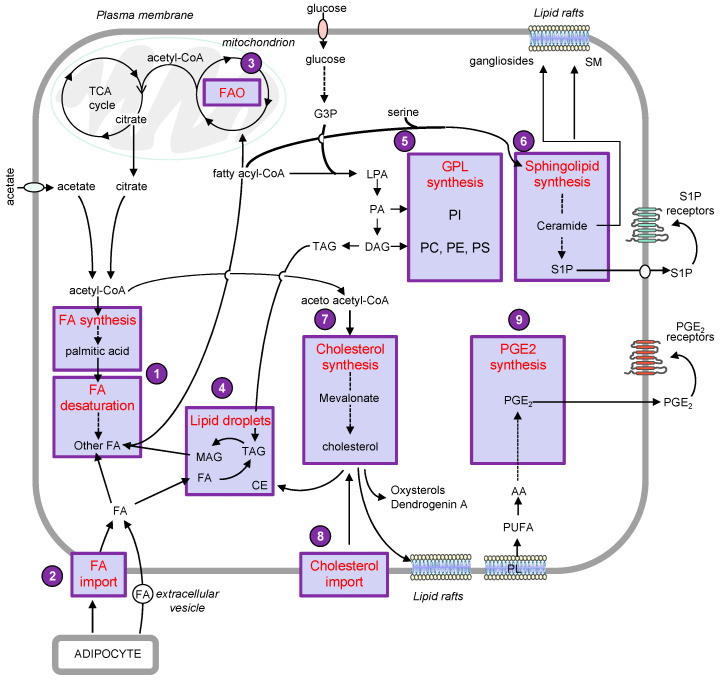
Schematic overview of the lipid metabolic network that regulates melanoma progression. The figure highlights the lipid pathways that are mostly altered in melanoma cells: (**1**) the de novo synthesis, elongation and desaturation of fatty acids (FA), which produce the repertoire of FA with different saturation levels. (**2**) The import of FA from neighboring adipocytes that can fuel FA β-oxidation (FAO) in mitochondria (**3**) to produce energy. (**4**) The lipid droplets, composed of neutral lipids, i.e., triacylglycerol (TAG) and cholesteryl ester (CE), which are critical to melanoma cell aggressiveness. (**5**) The synthesis of glycerophospholipids (GPL), including phosphatidylcholine (PC), phosphatidylethanolamine (PE,) phosphatidylserine (PS) and phosphatidylinositol (PI), which are produced from glycerol-3-phosphate (G3P). (**6**) The synthesis of sphingolipids, which begins with the condensation of serine and FA-Coenzyme A conjugates. Sphingolipids and glyceroPL are precursors of lipid mediators involved in cell signaling pathways and are used to build cell membranes in order to sustain cancer cell proliferation. (**7**) The cholesterol biosynthesis, initiated by the conversion of acetyl-CoA to acetoacetyl-CoA, and (**8**) the cholesterol import from the bloodstream. Cholesterol and sphingolipids, i.e., sphingomyelin (SM) and gangliosides, are part of the lipid rafts, which act as signaling hubs in cancer cell proliferation, adhesion and migration. (**9**) The synthesis of prostaglandin E2 (PGE2) from arachidonic acid (AA), a long-chain polyunsaturated FA (PUFA) freed from phospholipids (PL). PGE2 and the sphingolipid metabolite S1P are secreted and act through cell surface receptors to suppress immune response and promote melanoma progression. Abbreviations: LPA, lysophosphatidic acid; MAG, monoacylglycerol; PA, phosphatidic acid; TCA, tricarboxylic acid.

Interestingly, emerging evidence reported numerous alterations of the lipid metabolic network that could sustain cell growth and metastasis in melanoma cells (Figure 1).

The most prominent phenomenon is an increased rate of lipogenesis, in which nutrient-derived carbons get converted into fatty acids (FAs), sterols and complex lipids. Lipogenesis relies mainly on the availability of acetyl-CoA. The main precursor of cytosolic acetyl-CoA is citrate originating from the tricarboxylic acid (TCA) cycle under normal conditions [4]. This conversion is catalyzed by ATP citrate lyase (ACLY), which is overexpressed in a variety of cancer types, including melanoma. Moreover, increased ACLY expression was associated with poor outcome of patients with melanoma [5,6]. During metabolic stress such as hypoxia, the synthesis of acetyl-CoA preferentially originates from acetate [7,8]. Acetate dependence is specific to BRAF mutant but not NRAS mutant or wild-type BRAF/NRAS melanoma cells [9]. Importantly, melanoma brain metastases, which are associated with an extremely poor prognosis, have been shown to exhibit increased dependency on acetate [10].

FA synthesis starts with the carboxylation of acetyl-CoA to malonyl-CoA, which is catalyzed by acetyl-CoA carboxylase (ACC1). Then, through a series of repetitive condensations catalyzed by the FA synthase (FASN), molecules of malonyl-CoA are assembled to form palmitic acid. The expression of ACC1 [11] and FASN [12] is upregulated in human melanoma, as compared to conventional nevi. The increased expression of FASN occurs independently of the *BRAF* and *NRAS* mutation status [5] but is associated with the Breslow thickness and poor prognosis [12,13]. The specific inhibition of FASN activity with the anti-obesity drug Orlistat was reported to reduce the occurrence and number of lung metastases in a murine model of melanoma [14]. Thereafter, elongation and desaturation of palmitic acid generate the basis for a diverse spectrum of saturated and unsaturated FA that can be activated into fatty acyl-CoA by acyl-CoA synthetase long-chain (ACSL) family members. Of note, the expression of ACSL3 has been also associated to a worse prognosis in melanoma [15]. Moreover, a recent study reported that oleic acid, an abundant FA in lymph, protected melanoma cells from ferroptosis in an ACSL3-dependent manner and increased their capacity to form metastasis [16]. Once activated, the FA can be incorporated into triglycerides (also named triacylglycerols (TAGs)), glycerophospholipids (GPL) and sphingolipids (SL) or undergo β-oxidation in mitochondria for energy generation [17]. In addition to their role in fueling various lipid metabolisms, FAs also participate to protein acylation, thereby controlling protein trafficking, membrane localization and signaling activities [18]. For instance, the S-palmitoylation of the melanocortin-1 receptor (MC1R), which corresponds to the covalent attachment of palmitic acid to the protein at cysteine residues, was associated with MC1R activation, thereby reducing melanomagenesis in mice [19]. Conversely, the S-palmitoylation of the TEA domain (TEAD) transcription factors was shown to be critical in TEAD’s binding to the Hippo kinases YAP (Yes-associated protein) and TAZ (Transcriptional activator with PDZ domain) [20]. The YAP/TAZ-TEAD complex is known to activate expression of several genes that favor tumor growth and metastasis in various solid cancers, including melanoma [21].

Beside FA synthesis, the cytosolic acetyl-CoA can also be transformed into 3-hydroxy-3-methylglutaryl-CoA (HMG-CoA), which is then converted into mevalonate by the HMG-CoA reductase (HMGCR), the rate-limiting step of cholesterol biosynthesis. Analysis of public databases revealed that ~60% of melanomas had increased expression (including chromosomal copy number increases) in at least one of the cholesterol synthesis genes. These events were associated with decreased melanoma patient survival [22].

While de novo lipogenesis constitutes a valuable source of energy, as well as lipid mediators, hypoxia or driver mutations can also prime melanoma cells to consume FA from the TME, via FA β-oxidation (FAO), to meet their energetic demands [23]. FAO was reported to promote melanoma progression. For instance, carnitine palmitoyltransferase 2 (CPT2), which is critical for translocation of long-chain acyl-CoA into the mitochondrial matrix, is one of the most significantly upregulated genes in melanoma as compared to benign nevi [24]. Moreover, thanks to a targeted analysis of human tumor samples from the TCGA database, it was recently revealed that increased expression of FAO enzymes correlated with poor overall survival in melanoma patients [25]. In accordance, it was demonstrated that FAO contributed significantly to the energy reserves of metastatic 4C11+ cells, which were derived from melan-a melanocytes after sequential detachment-re-adhesion cycles [26]. How FAO promotes melanoma progression is still unclear. One can imagine that FAs serve as a valuable source of acetyl-CoA that contributes to citrate formation, after entering the TCA cycle, and provide an ATP boost for tumor cells under nutrient-depleted conditions [27]. Interestingly, other studies in which melanoma cells were co-cultured with adipocytes have shown that adipocyte-derived lipids were utilized in the FAO pathway and decreased the dependence on de novo lipogenesis [25,28]. In this context, glucose oxidation and lactate release were unchanged, indicating that glycolysis was not impacted [29].

FA can be imported from plasma and lymph [16] either through the FA translocase (FAT/CD36), the plasma membrane-associated FA binding proteins (FABP) or the FA transport proteins (FATP), and FA levels can increase through close contact with adipocytes [28,30]. This latter event was inhibited by the FATP inhibitor lipofermata in a zebrafish melanoma model [28]. Importantly, a recent study reported that, when melanoma cells were exposed to the aged fibroblast lipid secretome, they increased FA uptake via FATP2, whose expression was upregulated. Inhibiting FATP2 with lipofermata was shown to overcome age-related resistance to BRAF/MEK inhibition in animal models and significantly extend survival in older animals [31]. Moreover, FABP7 has been associated with increased proliferation and invasive properties of melanoma cells [32,33,34]. CD36-mediated FA uptake is prominent in metastasis-initiating melanoma cells, and this change was correlated with poor prognosis in melanoma patients [35], thereby highlighting the importance of FA uptake for melanoma progression.

Increased FA biosynthesis and FA uptake may lead to increased levels of multiple lipids with a signaling function that can affect numerous cellular processes, including melanoma cell differentiation and motility. Melanoma is notorious for its high metastatic potential. Melanoma invasive behavior is controlled by signaling pathways, e.g., the canonical and non-canonical Wingless-type (Wnt) and the transforming growth factor beta (TGF-β) signaling pathways, that have been described to not only regulate the actin cytoskeleton but also the expression levels and the function of the lineage commitment factor microphthalmia-associated transcription factor (MITF) [36]. A wide range of cellular stresses including hypoxia [37,38,39], low glucose [40] and inflammatory signaling [41,42,43] were shown to reduce MITF expression and increase the metastatic properties of melanoma cells. Moreover, meta-analysis of gene expression profiling of hundreds of human melanoma cells identified a highly invasive phenotype, characterized by extremely low MITF expression, associated with a stemness- and epithelial-to-mesenchymal transition (EMT)-based gene expression signature [44,45,46]. It is now well recognized that melanoma cells are highly plastic and can undergo phenotype switching that contributes to tumor progression. During this process, melanoma cells with an MITF-low phenotype undergo invasion and dissemination, and then switch back to an MITF-high phenotype at the metastatic site in order to proliferate [47]. Importantly, the reduction of MITF expression has been associated with a switch in EMT-associated transcription factors (EMT-TFs). In particular, a reduced expression of ZEB2 and SNAIL2, in favor of an increased expression in ZEB1 and TWIST1, has been linked to MITF downregulation, E-cadherin loss and increased invasive properties of human melanoma cells [48].

Interestingly, recent findings revealed that the lipogenic enzyme ACLY regulated MITF, and its downstream transcriptional targets by controlling histone acetylation at its promoter [6]. Moreover, low activity of stearoyl-CoA desaturase (SCD), which catalyzes the rate-limiting step of FA desaturation, reduced MITF expression and maintained melanoma cells in an MITF-low de-differentiated state [49]. Inversely, MITF was identified as a regulator of SCD expression and FA saturation, thereby establishing a positive feedback loop to stabilize an MITF-low state associated with increased metastatic dissemination. Mechanistically, low SCD expression and activity promoted ER stress and the phosphorylation of eukaryotic initiation factor-α (eIF2α) leading to the activation of an ATF4- and NF-κB-dependent inflammatory signaling that sustains a reduced MITF expression and melanoma cell dedifferentiation [49]. These data demonstrate that FA metabolism can regulate melanoma cell differentiation and progression.

**Figure 2:**
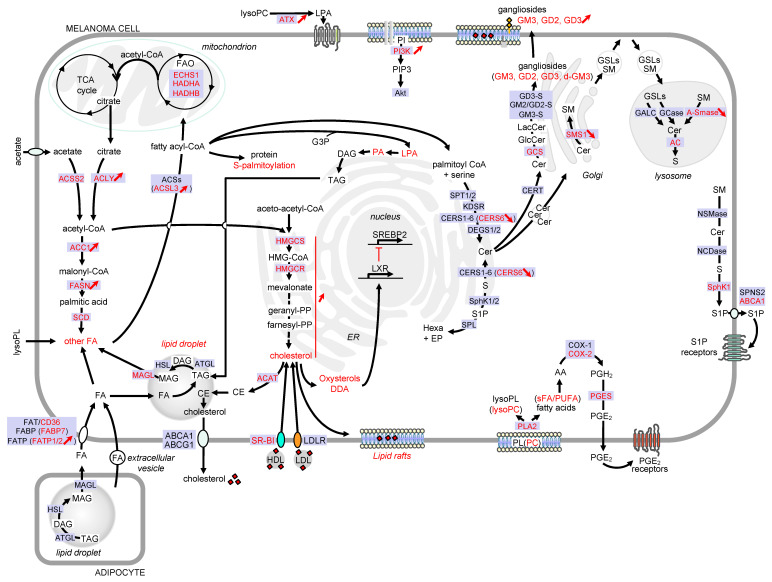
Detailed view of the major alterations of lipid storage and metabolism pathways during melanoma development. Only lipid pathways with reported modifications in melanoma are represented. Enzymes, receptors and transporters are indicated in blue boxes. Modifications in melanoma are highlighted in red. Abbreviations: AA, arachidonic acid; ABC, ATP-binding cassette transporter; AC, acid ceramidase; ACAT, acyl-CoA: cholesterol acyltransferase; ACC, acetyl-CoA carboxylase; ACLY, ATP citrate lyase; ACS, acyl-CoA synthetase; ACSL3, acyl-CoA synthetase long chain 3; Akt, AKT serine/threonine kinase; A-SMase, acid sphingomyelinase; ATX, lysophospholipase D autotaxin; CE, cholesteryl ester; Cer, ceramide; CERS, ceramide synthase; CERT, ceramide transport protein; COX, cyclooxygenase; DAG, diacylglycerol; DDA, dendrogenin A; DEGS, dihydroceramide desaturase; d-GM3, de-N-acetyl GM3; ECHS1, enoyl-CoA hydratase short chain 1; EP, ethanolamine 1-phosphate; ER, endoplasmic reticulum; FA, fatty acid; FAO, fatty acid -oxidation; FASN, fatty acid synthase; FAT, fatty acid translocase; FABP, fatty acid binding protein; FATP, fatty acid transport protein; GALC, galactosylceramidase; GCase, glucosylceramidase; GCS, glucosylceramide synthase; GD3-S, GD3 synthase; GM2/GD2-S, GD2/GM2 synthase; GlcCer, glucosylceramide; GM3-S, GM3 synthase; GSL, glycosphingolipid; G3P, glycerol-3-phosphate; HADHA, hydroxyacyl-CoA dehydrogenase subunit alpha; HADHB, hydroxyacyl-CoA dehydrogenase subunit beta; hexa, hexadecenal; HMG-CoA, 3-hydroxy-3-methylglutaryl-CoA; HMGCR, HMG-CoA reductase; HMGCS, HMG-CoA synthase; KDSR, 3-ketosphinganine reductase; LacCer, lactosylceramide; LDL, low-density lipoprotein; LDLR, low-density lipoprotein receptor; LPA, lysophosphatidic acid; lysoPC, lysophosphatidylcholine; lysoPL, lysophospholipid; LXR, liver X receptors; MAG, monoacylglycerol; MAGL, monoacylglycerol lipase; NCDase, neutral ceramidase; NSMase, neutral sphingomyelinase; PA, phosphatidic acid; PC, phosphatidylcholine; PGE2, prostaglandin E2; PGES, prostaglandin E synthase; PGH2, prostaglandin H2; PI, phosphatidylinositol; PI3K, phosphatidylinositol-3-kinase; PIP3, phosphatidylinositol-3,4,5-triphosphate; PL, phospholipid; PLA2, phospholipase A2; PUFA, polyunsaturated fatty acid; S, sphingosine; SCD, stearoyl-CoA desaturase; sFA, saturated fatty acid; SM, sphingomyelin; SMS, sphingomyelin synthase; SphK, sphingosine kinase; SPL, sphingosine 1-phosphate lyase; SPNS2, sphingolipid transporter 2; SPT, serine palmitoyltransferase; SR-BI, scavenger receptor class B type I; SREBP2, sterol regulatory element binding protein 2; S1P, sphingosine 1-phosphate; TAG, triacylglycerol; TCA, tricarboxylic acid.

This review aims to illustrate the major alterations affecting lipid storage organelles and the metabolism of the main lipid classes during melanoma development (see Figure 2 for a detailed view) and how these metabolic dysregulations contribute to phenotype plasticity and/or melanoma aggressiveness. How these metabolic vulnerabilities could be targeted for therapeutic benefit is also highlighted.

## 2. Lipid Droplets

Lipid droplets (LD), also referred to as adiposomes, are the major cellular organelles for the storage of neutral lipids, such as cholesteryl ester (CE) and TAG. TAGs are sequentially hydrolyzed, by three different lipases, into free FA that can be mobilized for energy production, membrane synthesis and generation of essential lipid-derived molecules. A highly aggressive behavior of melanoma cells has been associated with increased expression of monoacylglycerol lipase (MAGL), that catalyzes the hydrolysis of monoacylglycerols into FA and glycerol, and MAGL inhibition was shown to reduce melanoma cell migration and survival [50]. MAGL has been identified in an EMT-associated gene signature in solid cancers [51], and its upregulation resulted in cancer progression via NF- κB-mediated EMT [52].

A comparative analysis of LD composition has recently revealed that the dedifferentiated melanoma cell line M381 exhibited a relatively increased level of unsaturated CE and TAG, as compared to more differentiated melanoma cell lines [53]. Importantly, a prolonged treatment with the SCD1 inhibitor CAY10566 resulted in decreased levels of unsaturated lipids within LD and, inversely, in an excess of saturated FA that can modify membrane fluidity and lead to apoptosis [53]. The SCD1 inhibitor A939572 was also shown to prevent the proliferation of the MITF-high/proliferative IGR37 and 501mel melanoma cell lines [49]. However, in this latter study, the authors demonstrated that the SCD1 inhibitor was not effective in the MITF-low/invasive IGR39 and A375M melanoma cell lines. Moreover, consistent with the differential effect of SCD1 inhibition on the MITF-high and MITF-low cell lines, the SCD inhibitor A939572 only substantially increased the saturated FA: monounsaturated FA ratio in the sensitive IGR37 cell line, but not in the insensitive IGR39 cell line. These discordant observations emphasize the importance of investigating the impact of MITF expression and/or melanoma cell phenotype on LD composition.

It is recognized that LD accumulation is induced by hypoxia in an HIF-1α-dependent manner and is associated with increased FABP-mediated FA uptake [54]. Interestingly, a lipid storing-phenotype with LD accumulation, was observed in a melanoma stem cell (MSC) model as compared to differentiated melanoma cells [55]. In accordance, the metastatic potential of human FEMX-1 melanoma cells has been reported to correlate with LD enrichment [56]. Of note, a high-content genome-wide RNAi screen revealed that Wnt ligands can potently promote LD accumulation [57]. Wnt5A-mediated activation of STAT3 was shown to reduce MITF levels and ultimately downregulates the expression of melanocyte differentiation antigens [58]. However, it is currently not known if a reduced expression of MITF is associated with LD accumulation in dedifferentiated melanoma cells.

Interestingly, studies also reported that FA produced by neighboring adipocytes, which were transferred to melanoma cells through extracellular vesicles (EVs), were stored in LD as TAG, and potentially hydrolyzed by lysosomal lipases to fuel FAO. This phenomenon led to the relocalization of mitochondria, as well as LD and lysosomes, to membrane protrusions and was associated with tumor cell migration [25]. Accordingly, the EV-mediated increase in melanoma cell migration was abrogated by the FAO inhibitors etomoxir and trimetazidine [29] and the mitochondrial fission inhibitor Mdivi-1 [25].

Altogether, these observations reveal that melanoma cells can utilize LD accumulation and metabolism to support their malignant behavior.

## 3. Phospholipids

GPL and some SL constitute the main phospholipids. GPLs are glycerol-based phospholipids, whereas SL refer to a class of complex lipids containing a sphingoid long-chain base (amino alcohol), which is synthesized from serine and a long-chain fatty acyl-CoA. Together, GPL and SL represent the major structural components of biological membranes and are also a source of biologically active compounds.

### 3.1. Glycerophospholipids: Potential Roles in Melanoma Progression and Therapeutic Approaches

GPL are composed of 1,2-diacylglycerol and a phosphodiester bridge linking the glycerol backbone to a polar headgroup, such as choline, serine, ethanolamine, inositol or glycerol, to form phosphatidylcholine (PC), phosphatidylserine (PS), phosphatidylethanolamine (PE), phosphatidylinositol (PI) or phosphatidylglycerol (PG), respectively. Interestingly, it was reported that melanoma-derived microvesicles favor the establishment of metastasis in a PS-dependent manner, possibly by downregulating the host’s inflammatory immune responses [59]. Moreover, lipidome analyses revealed aberrant GPL metabolism with increased levels of PE and PC species in zebrafish V12RAS-driven melanoma [60]. Importantly, several studies have shown that cancer cells undergoing EMT have increased PC content and that phosphatidic acid (PA), a key intermediate metabolite in the synthesis of GPL, could maintain the stemness of cancer cells, by reducing apoptosis [61]. Moreover, lysoPA (LPA), which is produced by the phospholipase A2 (PLA2)-catalyzed deacylation of PA, was reported to stimulate melanoma invasion in 2D and 3D assays [62] and to induce MITF degradation [63].

In contrast, melanoma cells display a high ability to hydrolyze lysoPC (LPC), which derives from the cleavage of PC via the action of PLA2 [64]. Indeed, LPC inhibits the formation of focal adhesion complexes, affects integrin activity and thereby reduces the metastatic spread of melanoma cells, as shown in the B16F10 murine melanoma model [65]. LPC may be converted to LPA by the lysophospholipase D autotaxin (ATX). Interestingly, high expression of ATX promotes melanoma motility/invasiveness and enhances in vivo metastatic potential [66].

Furthermore, PI species with saturated and monounsaturated FA chains were reported to increase with tumor stage in murine [67] and human melanoma [68]. A previous lipidomic study showed that the ratio of saturated to unsaturated FA increased in highly metastatic B16F10 cells, as compared to poorly metastatic B16F1 cells, suggesting that membrane fluidity could, counterintuitively, decrease during melanoma progression [69]. PI metabolism leads to the formation of phosphatidylinositol-3,4,5-trisphosphate (PIP3) through the phosphatidylinositol-3-kinase (PI3K). PIP3 can be dephosphorylated by the lipid-phosphate PTEN into PIP2. However, genetic inactivation of PTEN is frequently found in melanoma [70], limiting this transformation. In contrast, the expression of the p85 subunit of PI3K was higher in metastatic melanoma relative to primary melanoma as observed in a large cohort study [71]. The activation of the PI3K/AKT pathway has been shown to suppress transcription of the cell adhesion molecule E-cadherin, thereby leading to a more invasive phenotype of melanoma cells [72]. Accordingly, PTEN inactivation, which results in the serine threonine kinase AKT activation, decreased E-cadherin in RAS-activated melanoma cells [73]. Moreover, increased expression of AKT was associated with a poor five-year melanoma-patient survival rate [74]. Interestingly, AKT could promote MITF degradation [75], therefore affecting its differentiation-associated functions.

### 3.2. Sphingolipids

SLs consist of an 18-carbon amino alcohol backbone, usually sphingosine, to which an FA may be attached through an amide bond, and a headgroup at the primary hydroxyl. Types of SLs include simple SL, e.g., ceramide, sphingosine and sphingosine 1-phoshate (S1P), and more complex SLs, such as sphingomyelin (SM), glycoSL and gangliosides. Numerous studies showed that SL metabolism is dysregulated in melanoma cells, in order to reduce the intracellular level of ceramide, which is known to promote apoptosis (for review, see References [76,77]). This results in the accumulation of metabolites having a pro-tumoral action, including the ceramide derivatives S1P and gangliosides. Here, we report studies demonstrating that several SL metabolites or SL-metabolizing enzymes play a critical role in the melanoma cell phenotypic switch, as well as in melanoma progression.

#### 3.2.1. Potential Roles of Sphingolipids in Melanoma Progression

##### Role of Ceramide and S1P in Melanoma Progression

Previous studies revealed that, whereas SL-metabolizing enzymes promoting ceramide formation were downregulated during melanoma progression, those responsible for ceramide degradation were upregulated [77]. For instance, a low expression of the ceramide synthase CerS6 was associated with the invasive capacities of different human melanoma cells [78]. Moreover, the expression of the acid sphingomyelinase (A-SMase), which hydrolyzes SM into ceramide, was lower in primary melanomas than in benign nevi, and further reduced in the lymph-node metastases. As a matter of fact, in vitro and in vivo measurements of invasion demonstrated that the expression of A-SMase was negatively correlated with melanoma aggressiveness [79]. Unexpectedly, a lower expression/activity of A-SMase was observed in hyper-pigmented murine and human melanomas, as compared to the hypo-pigmented ones, suggesting an inverse correlation between A-SMase expression/activity and melanin content. Mechanistically, A-SMase was proposed to induce ERK-mediated MITF degradation by the proteasome, associated with a downregulation of MITF targets CDK2, Bcl-2 and c-MET [79]. In accordance, increasing the intracellular content of ceramide in human melanoma cells by blocking its conversion into glucosylceramide (GlcCer) with the GlcCer synthase inhibitor PDMP [80] or by adding short-chain C2-ceramide [81], reduced cell proliferation or motility, depending on cell lines. Of note, C2-ceramide was shown to reduce MITF expression in human melanocytes, suggesting that ceramide could affect the phenotype switching associated with melanoma progression [82].

It was recently provided evidence that *SGMS1*, the gene encoding SM synthase 1 (SMS1), is frequently downregulated in melanoma and SMS1 downregulation is associated with a bad prognosis in metastatic melanoma patients. SMS1 triggers the ceramide conversion to SM [83]. However, human melanoma cell lines exhibiting low SMS1 expression do not accumulate intracellular ceramide. Rather, they display higher intracellular levels of GlcCer than SM and ceramide, suggesting that ceramide is mostly metabolized towards the GSL pathway. Whether and how SMS1 downregulation and the consequent SL metabolism alterations modulate MITF and melanoma progression remains to be investigated [83].

Of interest is the finding that degradation of ceramide into sphingosine via the acid ceramidase (AC), followed by phosphorylation of sphingosine to S1P, by sphingosine kinase 1 (SphK1), is associated with melanoma progression [77]. AC was shown to be highly expressed in melanocytes and proliferative melanoma cells in vitro, as well as in biopsies from patients with stage II melanoma [84]. It was recently demonstrated that MITF expression increased in AC-overexpressing melanoma cells and observed that AC expression was higher in human melanoma cells exhibiting a proliferative phenotype as compared to invasive ones [85]. In contrast, AC-knockout A375 cells, which accumulate long-chain saturated ceramides, showed a strong decrease of MITF expression, as well as MYC, CDK1, CHK1 and AKT, and were blocked at the G1/S cell-cycle checkpoint [86]. Downregulation of AC in melanoma cells was also reported to induce E-cadherin loss and, inversely, to increase expression of TWIST1, which is in accordance with a more aggressive phenotype [84]. In addition, it was demonstrated that low AC expression was associated to increased FAK phosphorylation and relocation at focal adhesions. This phenomenon led to increased expression of integrin β5 and integrin αV, which play a critical role in the invasive behavior of melanoma cells [85]. Finally, using a ChIP-Seq database, AC was identified as a direct target of MITF, demonstrating that MITF and AC are part of a positive feedback loop that controls melanoma plasticity [85].

Consistent with the high levels of SphK1 reported melanoma cells [87,88,89], a shift of the S1P-ceramide balance towards S1P production was observed. SphK1 activity is induced by ERK1/2 [87,88] and SphK1 knockdown impaired anchorage-dependent and -independent growth of different human melanoma cells in vitro [87], as well as B16F10 [90] and Yumm1.7 [89] murine melanoma cell growth in vivo. S1P conveys oncogenic signals mainly through five G-protein coupled receptors, named S1P receptors (S1P1-5), which are expressed both on tumor cells and neighboring cells in the TME [91]. A previous study reported that S1P can activate cell migration in S1P1-overexpressing B16F10 cells and, inversely, inhibit melanoma cell migration in S1P2-expressing cells, with the concomitant inhibition of the small GTPase Rac and stimulation of RhoA, demonstrating a receptor subtype-specific action of S1P in these cells [92]. Importantly, the SphK1/S1P pathway could also modulate MITF levels, probably by acting on signaling pathways known to regulate its expression in melanoma cells. Many studies reported that the SphK1/S1P axis was associated with the TGF-β signaling, which is known to repress MITF expression through the transcription factor GLI2 [93], promoting maintenance of melanocyte stem cells in a quiescent state [94]. Actually, S1P was shown to increase TGF-β expression and secretion in different cancers, including melanoma [90]. Moreover, through its binding to S1P receptors, S1P was able to induce TGF-β receptor trans-activation, resulting in SMAD phosphorylation and cell migration [95,96]. Inversely, TGF-β stimulated SphK expression, thereby controlling TGF-β-mediated extracellular matrix (ECM) remodeling, cell migration and invasion [97,98]. In addition, FTY720-induced SphK1 inhibition was associated with reduced β-catenin expression [99]. The Wnt/β-catenin pathway is a well-known activator of MITF expression in melanoma cells [100], and deactivation correlates with a higher metastatic potential [44], suggesting a role of the Sphk1/S1P axis in melanoma aggressiveness through MITF downregulation via the Wnt/β-catenin pathway.

##### Role of Gangliosides in Melanoma Progression

Several studies have revealed the role of gangliosides in melanoma progression [77]. Gangliosides are sialic acid-containing glycoSLs that are also named oligoglycosylceramides derived as a first step from lactosylceramide. High amounts of gangliosides, especially GM3, GD2 and GD3, are present in human melanoma cells and tissues (for review, see Reference [101]). Melanoma cells that overexpress GD3 synthase showed increased proliferative capacities [102], whereas those treated with the anti-GD3 antibody R24 had reduced growth in vitro and in mice [103]. Mechanistically, GD3 has been shown to mediate melanoma cell proliferation through the convergence of pro-tumoral signals such as hepatocyte growth factor (HGF) and the c-MET receptor tyrosine kinase [104]. GD3 also favors adhesion of GD3 synthase-overexpressing melanoma cells to the ECM by recruiting integrins through glycolipid-enriched microdomains [105]. Similarly, overexpression of GD2 synthase promoted melanoma cell adhesion [106]. Finally, GD3 was reported to stimulate melanoma cell invasion via p130Cas or paxillin, which are key components of the focal adhesion cytoskeleton [102]. More recently, it was demonstrated, using GD3-high or GD2-high melanoma cells, that GD2 enhanced the adhesion properties of melanoma cells, while GD3 stimulated their invasive capacities [106]. These observations unveil a critical role for gangliosides in the switch from proliferative to migratory phenotypic states, with GD2 acting at the primary and metastatic sites, in order to promote cell proliferation, and GD3 as a potential inducer of melanoma cell invasion, in order to reach a metastatic niche [106]. Interestingly, the addition of the GD3 precursor GM3 only to B16 melanoma cells with low metastatic potential to lungs increased their dissemination in mice [107]. Moreover, de-N-acetyl GM3 (d-GM3), a derivative of ganglioside GM3, was mainly found in metastatic melanomas but not in benign nevi or most primary melanomas. d-GM3 containing melanoma cells possess increased migratory and invasive capacities, as compared to melanoma cells lacking d-GM3, thanks to the stimulation of MMP2 expression via the urokinase-like plasminogen activator receptor [108].

Whether gangliosides promote a pseudo-EMT in melanoma cells remains to be evaluated; however, numerous studies have demonstrated that glycoSL-metabolizing enzymes are connected with EMT-TFs in different cancer cells [109]. For instance, an increased expression of GD3 synthase was reported in transformed human mammary epithelial cells overexpressing TWIST1 or SNAIL1 [110]. GD3 synthase knockdown reduced breast-cancer stem-cell-associated properties and completely abrogated tumor formation in vivo. Otherwise, inhibition of GlcCer synthase significantly decreased the expression of ZEB1 and β-catenin in colon cancer stem cells [111].

Altogether, these studies clearly established that SL metabolism acts as a potential regulator of key actors of melanoma progression, opening novel therapeutic avenue for the prevention of metastasis.

#### 3.2.2. Therapeutic Approaches Targeting Sphingolipids in Melanoma

Due to their wide range of action in melanoma cells, SLs are emerging as a goldmine for new therapeutic agents, and the manipulation of their metabolism could be beneficial to control disease progression. Moreover, due to the propensity of melanoma cells to deplete ceramide by modifying the expression of AC, SphK1 and GlCer synthase, inhibitors targeting these enzymes have exhibited therapeutic potential by tipping the balance towards ceramide accumulation to promote cell death.

For instance, AC inhibition with the chemically stable inhibitor ARN14988, which increased ceramide levels, sensitized proliferative human melanoma cells to the cytotoxic action of various antitumor agents [84]. In accordance, it was previously reported that dacarbazine causes degradation of AC, and that this effect contributes to the drug′s cytotoxic action [112].

SphK1 inhibition was also proposed to decrease intracellular S1P levels and, inversely, to increase ceramide levels in cancer cells. Treatment of melanoma cells with the non-lipid pan-SphK inhibitor SKI-I led to cell cycle arrest at G0/G1 phase and apoptosis [87]. Moreover, the intraperitoneal administration of SKI-I in mice harboring melanoma decreased tumor growth [87,89,90]. Consistently, the growth of B16F10 tumors was impaired in SphK1^−/−^ mice as compared to wild-type animals [88]. SphK1 inhibitors also potentiated the inhibitory effects of commonly used antineoplastic drugs. Indeed, SphK1 inhibition by the immunomodulator FTY720, which is also a functional antagonist of S1P receptors, downregulated the PI3K/AKT/mTOR signaling pathways and EGFR expression in SK-Mel-28 and A375 human melanoma cells, resulting in an increased sensitivity to cisplatin [113]. Moreover, SphK1 inhibition, using either FTY720 or SKI-I in several melanoma cell lines, increased their sensitivity to the BRAF inhibitor vemurafenib [114,115]. Finally, SphK1 inhibition by the sphingosine-competitive inhibitor PF-543 [116] or Sphk1 downregulation by shRNA [89] enhanced the efficacy of immune checkpoint blockade therapies in murine melanoma models, reducing Treg induction and infiltration, respectively. In addition, Sphk1-deficient T cells display a high oxidative phosphorylation phenotype and capacity to produce IFN-γ and IL-17 upon TCR stimulation. Consequently, Sphk1-deficient T cells adoptively transferred into C57BL/6 mice are more efficient to control B16F10 melanoma growth as compared to wild-type T cells [102]. Thus, Sphk1 is a promising target for improving anti-melanoma immune response.

Inhibiting GlcCer synthase could be another way to increase intracellular levels of ceramide and to reduce those of gangliosides. The inhibition of GlcCer synthase by PDMP inhibited cell proliferation, migration and invasion of WM35 and WM451 human melanoma cells. These effects were associated with the inhibition of key enzymes from the glycolysis pathway, including the pyruvate kinase, the hexokinase and the lactic acid dehydrogenase [80]. Another GlcCer synthase inhibitor, the imino sugar OGT2378, decreased GM3 content and reduced MEB4 melanoma tumor burden in mice [117]. Importantly, gangliosides are expressed on the surface of melanoma cells and are considered as melanoma-associated antigens, that can be targeted in vaccination protocols. In a phase I study, 44% of patients with stage III or IV melanoma, who received GM3/VSSP vaccine, i.e., very small proteoliposomes containing GM3 ganglioside with *Neisseria meningitidis* outer membrane protein complex, showed an anti-GM3 IgM response with serum reactivity against melanoma cell lines and tumor biopsies [118]. Similarly, administration of a human IgM monoclonal antibody (L612 HuMAb), that binds GM3, led to an antitumor activity against melanoma in patients with stage IV melanoma [119].

Finally, introducing exogenous ceramides into cells has also been proposed as a method to trigger apoptosis in tumor cells. To this end, short-chain ceramides carried in pegylated nanoliposomes were used. Their administration to 1205Lu human melanoma cells reduced integrin affinity and impaired invasive capacities, via PI3K and PKCζ tumor-suppressive activities [120]. Moreover, in combination with sorafenib, nanoliposome containing ceramide inhibited growth of UACC-903 cells or 1205Lu cells xenografts, by targeting the MAPK and PI3K signaling pathways [121].

## 4. Sterols

Sterols belong to the isoprenoid family and cholesterol is the major sterol in mammalian tissues. Cholesterol plays a crucial role in membrane integrity and fluidity; in addition, as an essential component of lipid rafts, it also regulates endocytosis, membrane trafficking, cell signaling and motility. Biosynthesis of cholesterol, also called cholesterologenesis, occurs through the mevalonate pathway enzymes that condense three acetyl-CoA molecules in a two-step reaction to produce HMG-CoA. HMG-CoA is reduced to mevalonate by the HMGCR, the first rate-limiting enzyme in cholesterol biosynthesis. Then, a series of enzymatic reactions convert mevalonate to farnesyl pyrophosphate, that can be used to produce geranylgeranyl pyrophosphate for protein prenylation, as well as squalene for cholesterol synthesis (for review, see References [122,123,124,125]).

Cholesterol is synthesized de novo via HMGCR in melanocytes but exogenous cholesterol uptake can also occur via the LDL receptor (LDLR)/Apo-B100 pathway [126]. Cholesterol biosynthesis and uptake are tightly regulated in non-cancer cells by a mechanism of negative feedback that senses intracellular cholesterol concentrations [125]. Indeed, high cholesterol levels prevent activation of sterol regulatory element binding protein 2 (SREBP2), which, in addition to its involvement in FA synthesis, functions as a master transcriptional regulator of HMGCR and LDLR. A high cholesterol content also activates liver X receptors (LXRs), resulting in cholesterol synthesis inhibition, activation of cholesterol efflux via increased expression of ATP-binding cassette (ABC) transporters, and reduced uptake [122]. Finally, acyl-CoA-cholesterol acyl transferases (ACATs) can convert cholesterol into less toxic cholesteryl esters, that are usually stored in LD, or into oxidized derivatives, which will be ultimately metabolized into bile acids excreted by the digestive system.

### 4.1. Potential Roles of Sterols in Melanoma Progression

Cholesterol homeostasis is dysregulated in cancer cells, and changes in cholesterol metabolism substantially impact cancer progression, including cell proliferation, migration and invasion [122,127,128]. Such changes include increased cholesterol biosynthesis, increased exogenous cholesterol uptake by LDLR, elevated cholesterol esterification by ACAT1 and increased oxysterol production [124]. In melanoma, activation of the SREBP pathway and a positive feedback loop between SREBP-dependent lipogenesis and PI3K-AKT-mTORC1 signaling were shown to sustain growth of tumor cells in vitro and in vivo [129]. Of note, the activation of the SREBP pathway was independent of the oncogenic BRAF mutation. Analysis of TCGA data indicates that approximately 60% of melanoma samples display increased expression or chromosomal copy number in at least one of the cholesterol synthesis genes. Interestingly, overexpression of several of these genes was correlated with decreased melanoma patient survival [22]. In addition, the oxysterol 27-hydroxycholesterol was reported to promote melanoma cell proliferation by sustaining the AKT/MAPK signaling pathway [130], whereas pharmacological activation of LXRβ, the main isoform of LXRs expressed in melanoma cells, strongly inhibited tumor invasion and metastasis [131]. Collectively, these studies point to strong correlations between enhanced cholesterol metabolism and melanoma progression.

How changes in cholesterol metabolism participate in dedifferentiation and EMT-like process that sustain melanoma metastatic potency has been poorly investigated. Exogenous cholesterol increases melanogenesis in melanocytes and intermediate pigmented melanoma cells, via the production of cAMP, the subsequent activation of the CREB/MITF/tyrosinase pathway, and also presumably by stabilizing membranes and protecting melanogenic enzymes from proteasomal degradation. Unexpectedly, highest contents in cholesterol were found in melanosomes of amelanotic melanoma cells [126], suggesting a possible higher cholesterol demand in early stage melanosomes. The link between cholesterol levels and melanoma cell dedifferentiation has never been explored. Interestingly, the transient overexpression of CD271, a marker of dedifferentiated melanoma cells with stemness and invasive properties, induced the expression of genes involved in cholesterol synthesis [132]. In addition, the activation of LXRs by the synthetic ligand TO901317 potently inhibited melanogenesis through ERK-induced MITF degradation in human primary melanocytes and B16 melanoma cells [133]. Whether LXR receptors are involved in melanoma dedifferentiation during tumor progression remains unknown.

Noticeably, some proteins involved in cholesterol metabolism were shown to contribute to melanoma aggressiveness, independently of their known metabolic function. For instance, the scavenger receptor class B type I (SR-BI), which mediates the selective uptake of HDL cholesteryl ester into cells (including hepatocytes and steroidogenic cells) [134], was shown to drive an EMT-like phenotype in melanoma cells, independently of its cholesterol transporting function [135]. Gain- and loss-of-function of SR-BI revealed regulation of the proto-oncogene MET, an MITF target gene and a key driver of EV formation [136]. By enhancing the formation of EVs, SR-BI might contribute to the metastatic colonization. However, the potential role of SR-BI as an upstream regulator of MITF remains to be demonstrated. Nevertheless, different studies suggest a regulatory/feedback loop between SR-BI and MITF [126,137], in agreement with the strong correlation between their expression observed in melanoma patient samples [136].

Cholesterol and SL are essential components of cell membranes and are enriched in detergent-resistant membrane domains called lipid rafts, where major signaling processes, including those that control cancer cell survival and metastasis, take place [138]. Interestingly, recent findings showed that treatment of breast cancer cells with hydroxypropyl-β-cyclodextrin, a cholesterol-depleting agent of lipid rafts, inhibited the TGF-β/SMAD-induced EMT, based on increased expression of E-cadherin and decreased expression of vimentin [139]. Administration of methyl-β-cyclodextrin (MβCD), another cyclodextrin derivative, to melanoma-bearing mice, retarded tumor growth and extended animal survival. Mechanistically, MβCD was shown to block the protein kinase B/AKT (PKB) by inhibiting Src kinase and reactivating the negative PKB regulator, PP2A phosphatase [140]. In human melanoma cells, MβCD induced apoptosis [141] and affected cell morphology and migration [142]. More specifically, MβCD led to inactivation of Src by dissociation from lipid rafts, over-activation of RhoA, formation of robust stress fibers, inhibition of the internalization of β3 integrin and the dephosphorylation of the focal adhesion proteins paxillin and vinculin, resulting ultimately in the suppression of focal adhesion disassembly [142]. Moreover, cholesterol depletion significantly affects proton pumping activities of the V-ATPase, reducing the migratory and invasive capacities of B16F10 melanoma cells [143]. This proton pump helps maintaining an acidic TME that facilitates the activity of proteolytic enzymes, like metalloproteinases and cathepsins, thus creating a favorable microenvironment for migration and invasion [144]. Altogether, these studies show that cholesterol-containing lipid rafts are crucial to sustain cell morphology and the functions required for metastatic process in melanoma, although the underlying mechanisms are still unknown.

Cholesterol also exerts a key role in the formation and function of invadopodia, which are specialized cholesterol-rich membrane microdomains required for focalized ECM degradation. In human melanoma cells, invadopodia formation, function and structural integrity were shown to be dependent on plasma membrane cholesterol levels, as well as caveolin 1, a critical mediator of cholesterol transport to the plasma membrane [145].

Finally, it is also well recognized that cholesterol regulates membrane fluidity [146]. Recent findings demonstrated that motile cancer cells tend to exhibit lower membrane cholesterol levels to increase plasma membrane fluidity, which is essential to improve their ability to infiltrate various tissues [147,148]. Interestingly, in silico selected drugs for their putative inhibitory effects on EMT gene signature in breast cancer cells reduced cell membrane fluidity by increasing cholesterol levels. This resulted in decreased cell motility, stem cell-like properties and EMT in vitro, as well as metastasis inhibition in vivo, highlighting the importance of cholesterol in membrane fluidity and metastasis [149]. Unexpectedly, the ABC transporter ABCA1, which functions as a cholesterol reverse transporter, was shown to be expressed during EMT and drive metastatic properties in vitro and in vivo [149]. Finally, the association between cholesterol functions and cancer progression suggest a complex relationship between cholesterol and disease that is not yet fully understood.

### 4.2. Therapeutic Approaches Targeting Sterols in Melanoma

Targeting cholesterol metabolism was proposed to reduce cancer-related mortality. Indeed, the long-term administration of statins, e.g., atorvastatin, lovastatin, pivastatin or simvastatin, which are HMGCR inhibitors, was claimed to reduce the occurrence of different cancers [150,151], including melanoma [152,153]. This remains controversial [154], as the use of statins, in addition to systemic anticancer therapy, in patients with solid cancers did not improve overall survival or progression-free survival, as demonstrated by meta-analyses of clinical trials [155,156]. Nevertheless, statin use was associated with a reduced Breslow thickness [157], and a recent study suggests that statins may reduce recurrence in patients with high-risk melanoma, i.e., ulcerated primary melanoma [158].

At the cellular level, statins have been linked to the halting of melanoma cell-cycle progression [159,160] and the reduction of melanoma cell growth, migration and invasion [161], as well as the angiogenic activity of melanoma cells [162]. They also reduce tumor growth [160] and metastasis in mouse melanoma models [163,164,165], in part by abrogating the Rho/Rho-associated coiled-coil-containing protein kinase (ROCK) pathway [164]. Alterations in expression of matrix-metalloproteases and cytoskeletal reorganization may also contribute to the effects of the statins on invasion and migration of melanoma cells [161]. Of note, statin-sensitive cancer cell lines exhibit mesenchymal-like phenotypes characterized by abundant cytosolic vimentin and absent cell surface E-cadherin expression, while exogenous expression of cell surface E-cadherin converts statin sensitive cells to a partially resistant state [166].

Statins reduce the production of mevalonate and its downstream products, which have been shown to inhibit cancer cell growth and metastasis [167]. Interestingly, inhibition of the mevalonate pathway and consequently, of Rho-GTPase prenylation, stimulates melanoma immunogenicity [168] and leads to increased adaptive [169,170] and innate [165] immune response against the tumor [168].

Other compounds targeting cholesterol metabolism were proposed to control cancer growth. First, dendrogenin A (DDA) is a newly identified cholesterol derivative whose levels decrease in tumors, compared to normal tissues. DDA inhibited the cholesterol-5,6-epoxide hydrolase (ChEH) and was able to bind to LXRβ [171]. Interestingly, DDA complementation induced lethal autophagy in melanoma cells and reduced tumor growth in mice in an LXRβ-dependent manner [172]. Second, leelamine, a natural compound derived from the bark pine tree, was shown to delay melanoma growth in mice [173]. In vitro studies indicated that leelamine accumulates in acidic organelles such as lysosomes and inhibits the transport of cholesterol to the cytoplasm, leading to deficiency of free cholesterol [174]. As suggested by molecular docking analysis, leelamine presumably competes with cholesterol binding to the Niemann Pick type C protein type 1 (NPC1) protein, thereby affecting cholesterol export from the lysosome to the cytoplasm [175]. Lack of available cholesterol prevented endosome trafficking and receptor-mediated endocytosis, which in turn impaired receptor tyrosine kinase signaling and the activation of downstream PI3K/AKT, STAT3 and MAPK signaling pathways. Inhibition of these key oncogenic signaling by leelamine or the liposomal form of leelamine, nanolipolee-007 [176], decreased cell proliferation and, inversely, increased tumor cell apoptosis [173]. Nanolipolee-007 also reduced melanoma metastasis formation in spontaneous metastasis animal models, irrespective of the BRAF mutational status of the circulating tumor cells [177].

## 5. Eicosanoids

Eicosanoids are a class of bioactive lipids derived from 20-carbon polyunsaturated FA (PUFAs), most frequently arachidonic acid (AA). Eicosanoid biosynthesis is usually initiated by the activation of PLA2 family members that catalyze the hydrolysis of the sn-2 acyl bond of membrane GPL to produce free FA and lysophospholipids [178]. Several PLA2 isoforms, i.e., pla2g6, pla2g7 and pla2g10, appeared to be upregulated in zebrafish V12RAS-driven melanoma [60], and PLA2G6 gene was associated with melanoma risk in humans [179]. There are three major groups of eicosanoids formed via three distinct pathways: prostanoids, which include the prostaglandins and thromboxanes and are formed through the cyclooxygenase (COX) pathway, leukotrienes and related hydroxy FA coming from the lipoxygenase pathway, and epoxy and dihydroxy acids formed via epoxygenase (P450) pathways. Prostanoids and leukotrienes orchestrate complex interactions between cancer cells and the TME that govern cancer development and progression [180]. However, as the implication of leukotrienes in melanoma remains largely unexplored, we will focus on the role of the prostanoid pathway in melanoma progression.

### 5.1. Potential Roles of Prostanoids in Melanoma Progression

COX enzymes convert AA into prostaglandin H2 (PGH2), which is then transformed into prostaglandin E2 (PGE2) by the prostaglandin E synthase (PGES), encoded by the PTGES gene. COX enzymes exist in two isoforms COX-1 and COX-2 but PGE2 synthesis is mainly controlled by COX-2, which is encoded by the PTGS2 gene [181]. Elevated COX-2 expression is often associated with a poor prognosis in numerous cancers including melanoma [182,183,184] and has been linked to increased cell proliferation and invasion via activation of signaling pathways, playing a critical role in melanoma progression, such as the MAPK, the β-catenin and the EGFR/PI3K pathways.

High levels of COX-2 have been detected in both murine and human melanoma models [185,186]. However, conflicting results have been obtained regarding the expression of COX-2 in human melanoma. Vogt and colleagues observed that COX-2 is not expressed in benign and malignant melanocytic tumors [187], whereas Denkert et al. [186] have shown that it is expressed in primary melanoma cells, but not in benign nevi or in healthy epithelia. Another study showed that COX-2 expression is upregulated during melanoma progression, and consistently overexpressed in metastatic melanoma lesions [188]. In accordance, a recent study demonstrated that the level of COX-2 expression highly influences the metastatic ability of human melanoma cells independently of the presence of NRAS or BRAF mutations [189]. Furthermore, the modulation of COX-2 expression, either by gene disruption in mice, or using siRNA or specific COX-2 inhibitors in human cell lines, hindered the growth and invasiveness of melanoma cells [189]. B16 mouse melanoma cells injected into wild-type mice metastasized to bone and soft tissues, whereas tumor growth and metastasis were greatly diminished in Ptges^−/−^ mice. The authors showed that melanoma cells activate PGE2 signaling in stromal cells to support their progression, resulting in osteoclast activation, angiogenesis, and cancer cell dissemination [190]. While the pro-oncogenic mechanism of action of COX-2 in melanoma remains to be elucidated, these studies present clear evidence that COX-2 plays a key role in the progression of the disease.

Of note, TGF-β-induced EMT drives COX-2 expression, as well as PGE2 secretion, which in turn mediates cell migration and invasion through the PI3K pathway in prostate cancer cells [191]. Similar data were obtained in mammary epithelial cells, but this process depends on the inhibition of the SMAD2/3 pathway [192]. The COX-2/PGE2 signaling pathway itself can induce EMT in lung cancer cells via the activation of the β-catenin pathway [193]. However, even if the COX-2/PGE2 pathway drives dedifferentiation in various solid cancer cells, it seems to have an opposite role in melanoma cells. Indeed, PTGS2 silencing in human and murine melanoma cells resulted in decreased melanogenesis, as well as MITF expression [194]. Accordingly, treatment of B16F10 melanoma cells with the COX-2 inhibitor resveratrol decreased MITF expression via the MAPK and PI3K pathways [195]. Another study showed that ablation of Ptgs2 in B16F10 cells was associated with reduced cell proliferation, migration, and invasion in vivo [196]. Altogether, these results show that even though the COX-2/PGE2 pathway has been shown to promote EMT in different cancer cell types, it seems to enhance MITF expression in melanoma cells. Therefore, more data are needed to conclude whether this pathway favors an invasive or a proliferative phenotype in melanoma cells.

It is also well recognized that the COX-2/PGE2 pathway mediates immune suppression in melanoma. Indeed, genetic ablation of Ptgs2 in BrafV600E murine melanoma cells inoculated in mice was reported to stimulate the antitumor type I immunity [197]. Similarly, a recent study showed that Ptges knockdown in melanoma cells increased infiltration of CD8+ T and dendritic cells at the tumor site, leading to tumor growth inhibition [198]. These results are supported by data obtained from stage III melanoma patients for whom elevated PTGES expression was associated with low CD8+ T-cell infiltration, as well as poor patient survival [198]. To sum up, these data clearly illustrate the role that PGE2 plays to help cancer cells evade immune attacks and favor melanoma progression.

### 5.2. Therapeutic Approaches Targeting COX-2 in Melanoma

Until the discovery of the COX-2 isoform in the early 1990s, non-steroidal anti-inflammatory drugs (NSAIDs), e.g., aspirin, ibuprofen and naproxen, also called conventional NSAIDs, were effective inhibitors of both forms of COX. New NSAIDs, termed COXIBs for selective COX-2 inhibitors, were then developed. Both classical and selective NSAIDs demonstrated beneficial effects in preventing melanoma development [199,200] and progression [201] in humans, independently of sun exposure and age [199].

In mice, COX-2 inhibition limits cancer progression by promoting many effects, e.g., inhibition of tumor cell proliferation and invasion, stimulation of immune responses, limitation of cancer-associated inflammation or restriction of angiogenesis [184]. First, combination therapy based on IFN-γ and the selective COX-2 inhibitor NS-398 showed decreased B16F10 melanoma growth in syngeneic mice and improved survival as compared to IFN-γ alone [202]. NS-398 inhibited melanoma-induced suppression of macrophage functional activities. Second, ablation of *Ptgs2* in B16F10 cells was associated with reduced myeloid-derived suppressor cell differentiation in vitro, and inhibited tumor development and metastasis in vivo [196]. Third, the COX-2 inhibitor celecoxib induced apoptosis in melanoma cells through an oxidative stress [203]. Celecoxib also reduced the expression of programmed death-ligand 1 (PD-L1) [204] and indoleamine 2,3-dioxygenase 1 (IDO-1) [205], which are known to suppress antigen-presenting cells and cytotoxic cellular immune effectors in cancer [206,207]. Consequently, celecoxib was proposed as a valuable therapeutic adjuvant for melanoma treatment. In a phase II trial, metronomic cyclophosphamide and celecoxib have been added to a dendritic cell vaccine with the intent to dampen immunosuppressive mechanisms. The results showed that 6-month survival significantly increased compared to treatment without cyclophosphamide and celecoxib [208]. The results are still pending for another clinical trial (NCT03396952), which will assess the antiproliferative effect of aspirin in combination with monoclonal anti-PD-1 and anti-CTLA-4 antibodies in stage III and IV melanoma patients [184]. Finally, the dual COX-2/5-lipoxygenase inhibitor, licofelone, was shown to improve therapeutic melanoma vaccination by reducing immune-suppressive cell populations in mice bearing B16F10 melanoma cells [209]. Other examples of the use of NSAIDs in melanoma are summarized in Table 1.

## 6. Obesity and Melanoma: Role of Adipose Tissue in Tumor Progression

Lifestyle factors, such as diet and exercise and, consequently, the microbiote and obesity can alter lipid homeostasis in individuals but also influence melanoma development and progression (for a recent review, see References [211]). Only the impact of obesity, which is the most studied, is discussed here.

Epidemiological studies have shown that obesity is an established risk factor for melanoma incidence [212] and progression [213,214,215,216,217], even though several epidemiological studies indicated that this relation may be sex-dependent [212,218]. However, the correlation between melanoma and obesity also exists in premenopausal women [219] and when studies were adjusted for sunlight exposure [220,221,222], revealing that the differences first observed could in part be explained by self-limited sun exposure and menopausal status. Strikingly, a recent study has revealed that obesity is a good factor in term of response to targeted treatment or immunotherapy [223] and has been discussed as the “obesity paradox” [217]. This advantage was specific to males, possibly due to sex hormones or leptin immunosuppressive effects on T cell [223]. Preclinical studies have confirmed the positive correlation between obesity and melanoma size, lymph node involvement and lung metastasis [216].

Obesity is usually caused by an excessive accumulation of adipose tissue (AT), with this hypertrophic fat being the main driver of the pathologies associated with obesity, including cancer [224]. Several mechanisms linking obesity to melanoma have been described, including metabolic or endocrine processes (especially alterations in insulin/IGF1 signaling and sex hormone metabolism), inflammatory pathways and molecules secreted by adipocytes, the main cells of AT, such as leptin and adiponectin. As they have been discussed elsewhere [216,217], only lipid-dependent mechanisms are discussed here. As previously described, a preclinical study has revealed FA transfer between adipocyte and melanoma, which fuels melanoma metabolism through FAO, to promote melanoma aggressiveness [28]. Nevertheless, the impact of obesity has not been tested in this study. Melanoma cells can also internalize EV secreted by surrounding adipocytes [25,29,225]. The transfer of FAO enzymes via adipocyte EV drives melanoma cells towards a more aggressive phenotype. In the context of obesity, both effects were amplified due to the larger number of secreted EV, but also to the heightened FA content of individual EV [25].

Adipocytes can also secrete SL [226]. Interestingly, SphK1 expression in AT [227] and S1P levels in serum [228] were shown to increase with obesity; however, their role in the association between obesity and melanoma remains to be demonstrated.

Obesity is also characterized by chronic low-level elevation of inflammatory cytokines, such as IL-1, IL-6 and TNF-α, which can impair the immune system response and promote carcinogenesis [229,230]. Obesity is also associated with an increased infiltration of immunosuppressive cells into the tumor that sustain cancer progression [231]. Interestingly, adipose-derived stem cells have been shown to promote melanoma growth [232], revealing the role of proximal AT in the progression of this cancer. Moreover, obesity resulted in the activation of AT macrophages, a process still observable after weight loss [233]. These results revealed that the chronic inflammation induced by obesity could be considered as a part of trained immunity, a process that could be beneficial for patients and that could explain, at least in part, the obesity paradox observed in melanoma.

Whereas obesity clearly influences melanoma progression, further studies are needed to improve our understanding of the mechanisms orchestrating this complex interplay, and especially the role of lipids in this deleterious association.

## 7. Conclusions

The last few decades of work have revealed the importance of lipid metabolism in melanoma progression. Table 1 brings together studies in which a relationship between the expression of lipid-metabolism-associated genes and prediction of melanoma patient outcome has been demonstrated.

Moreover, the above-discussed studies highlight that targeting lipid metabolism may offer novel therapeutic strategies. Table 2 summarizes the in vitro and in vivo effects induced by pharmacological compounds known to target enzymes or receptors linked to lipid metabolism in different melanoma models.

The compounds listed in Table 2 that were included in clinical trials targeting melanoma are indicated in Table 3.

However, the large spectrum of functions lipid molecules fulfills in cell signaling underscores the importance of a more detailed understanding of the potential interplays between each lipid subfamily and the consequences in cancer progression. Understanding the link between alterations of the lipidome and the disease will also be useful for the development of novel lipid biomarkers. In this respect, it was recently showed that SphK1 expression constitutes a potential biomarker to predict melanoma progression and resistance to anti-PD-1 therapy. Indeed, it has been discovered that patients with low SphK1 expression in melanoma cells had significantly longer progression-free survival and overall survival than those with high SphK1 expression and patients with high SphK1 expression mostly failed to respond to anti-PD-1 therapy [89]. One might speculate that other modifications in lipid metabolism are potential biomarkers in melanoma; however, the discovery of clinically useful biomarkers still requires the inclusion of consistent large-scale proteomic studies in clinical trials.

## Figures and Tables

**Table 1:** Relationship between expression of lipid metabolism associated genes and prediction of melanoma patient outcome.

Lipid Classes	Genes	Expression in Melanoma	Outcome	References
FA	ACLY	Overexpressed	Worse prognosis	[5,6]
	ACSL3	Overexpressed	Worse prognosis	[15]
	CD36	Amplified in metastasis	Worse prognosis	[210]
	FAO (3 genes)	Overexpressed	Worse prognosis	[25]
	FASN	Overexpressed	Worse prognosis	[5]
	SCD	Overexpressed	Worse prognosis	[5]
SL	SGMS1	Downregulated	Worse prognosis	[83]
	SPHK1	Overexpressed	Shorter survival after anti-PD1	[89]
Sterols	Cholesterol synthesis (7 genes)	Overexpressed	Worse prognosis	[22]
Eicosanoids	COX-2	Overexpressed in primary melanoma	Decreased PFS; poor prognosis factors (thicker melanoma, high mitotic count)	[182,183]
	PTGES	Overexpressed	Worse prognosis (stage III melanoma)	[198]

PFS: progression-free survival.

**Table 2:** Effects of pharmacological agents targeting lipid metabolism in melanoma.

Targeted Enzyme/Receptor	Agent	Melanoma Cells/Models	Effects	Reference
FASN	Orlistat	B16F10	Reduced number of metastasis in mice	[14]
	Cerulenin		Reduced proliferation. Increased apoptosis	[234]
MAGL	JZL184	C8161	Decreased cell migration	[50]
SCD1	A939572	IGR37 501mel IGR39 A375M	IGR37, 501mel: Decreased cell proliferation Increased apoptosis IGR39, A375M: No effect	[49]
	CAY10566	M381	Apoptosis	[53]
FATP2	Lipofermata	WM793 1205Lu Yumm 1.7	Sensitization of melanoma cells in an aged TME to BRAF and MEK inhibitors Increased survival in old animals	[31]
FATP1		Zebrafish	Reduced melanoma growth and invasion	[28]
CPT1/ECHA (FAO)	Etomoxir/Trimetazidine	SK-Mel-28 1205Lu	Reduced melanoma migration	[29]
AC	ARN14988 ARN398	G361 A375	Sensitization of proliferative but not invasive cells to 5-FU	[84]
SphK1	FTY720	SK-Mel-28 A375	Increased cisplatin-induced apoptosis	[113]
		WM115 SK-Mel-28	Increased vemurafenib-induced apoptosis	[114]
	SKI-I	WM9	Reduced proliferation of vemurafenib-resistant cells	[115]
		Yumm 1.7	Increased efficacy of anti-PD1 and anti-CTLA-4 in mice	[89]
	PF-543	B16F10	Increased efficacy of anti-PD1 in mice	[116]
GCS	PDMP	B16F10	Increased genistein-induced apoptosis	[235]
	OGT2378	MEB4	Reduced tumor growth in mice	[117]
LXRβ	GW3965 T0901317	SK-Mel-2 SK-Mel-334.2 B16F10	Reduced cell invasion and sensitization to vemurafenib in vitro. Reduced tumor growth, angiogenesis and metastasis in vivo	[131]
ChEH LXRβ	DDA	B16F10, SK-MEL-28	Increased autophagy. Reduced tumor growth in mice	[172]
HMGCR	Simvastatin	A375M, G361, C8161, GAK, MMAc	Cell cycle arrest and increased apoptosis	[159]
		B16F10	Dose-dependent cell cycle arrest. Reduced tumor growth in mice	[160]
	Lovastatin, Mevastatin, Simvastatin	HT144, M14, SK-MEL-28	Reduced cell growth, migration and invasion	[161]
	Fluvastatin, Simvastatin	B16BL6	Reduced cell migration, adhesion and invasion in vitro and metastasis in mice	[164]
	Lovastatin	LB1319-MEL, BB74-MEL, LB2033-MEL, LB583-MEL	Increased expression of MHC class I Chain-related protein A (MICA)	[165]
		A375 and G361	Reduced cell growth and angiogenesis and increased apoptosis	[162]
	Atorvastatin	A375M, SK-MEL-28, WM-266-4	Reduced invasion in vitro and in mice	[163]
NPC1	Leelamine	Nine human melanoma cell lines	Reduced cell proliferation in vitro and tumor growth in mice	[173]
COX-2	NS-398	B16F10	Reduced cell growth and improved survival in mice	[202]
	Aspirin	Melanoma PDX cell lines A375, B16F10	Reduced cell motility, pigmentation in vitro, and tumor growth in immunodeficient mice	[236]
	Celecoxib	SK-Mel-5	Reduced cell proliferation	[189]
		B16F10	Increased ROS-dependent apoptosis	[203]
		KUL98-MELA	Rejection of IDO1-expressing human tumor xenografts in modified immunodeficient mice	[205]
		A375, SK-MEL-2	Reduced PD-L1 expression and cell growth	[237]
	Selenocoxib-1-GSH (analog of celecoxib)	WM35, WM115, WM278.1, A375M, 1205 Lu	Cell cycle arrest and increased apoptosis Reduced tumor growth in mice	[238]
	Celecoxib (+cyclophosphamide)	28 patients with metastatic melanoma	Six-month increase in survival	[208]
COX-2/5-lipoxygenase inhibitor	Licofelone	B16F10	Improved antitumor activity of a therapeutic melanoma vaccine	[209]

CPT1, Carnitine palmitoyltransferase I; ECHA, α subunit of the trifunctional enzyme.

**Table 3:** Clinical trials evaluating lipid-metabolism-targeting drugs in melanoma. Data were extracted from ClinicalTrials.gov database (https://clinicaltrials.gov).

Agent	Clinical Trial	Title	Posting Year	Status
Lovastatin	NCT00963664	Evaluation of interferon–lovastatin therapy for malignant melanoma	2009	Withdrawn
	NCT00462280	Lovastatin in treating patients at high risk of melanoma	2007	Completed (with results)
Fluvastatin	NCT04285749	Prevention of recurrence and metastasis in genetically high-risk melanomas	2020	Withdrawn
Aspirin	NCT04062032	Metabolomic and inflammatory effects of oral aspirin (ASA) in subjects at risk for melanoma	2019	Recruiting
	NCT04066725	Aspirin as an ultraviolet (UV) protectant in human subjects at risk for melanoma	2019	Recruiting
	NCT03396952	Prostaglandin inhibition and immune checkpoint blockade in melanoma	2018	Active, not recruiting
	NCT01753089	Dendritic-cell-activating scaffold in melanoma	2012	Active, not recruiting
Celecoxib	NCT04093323	Polarized dendritic cell (aDC1) vaccine, interferon alpha-2, rintatolimod, and celecoxib for the treatment of HLA-A2+ refractory melanoma	2019	Not yet recruiting
	NCT01313429	Tumor-cell vaccine for patients undergoing surgery for sarcomas, melanomas, germ cell tumors or malignancies that have metastasized to the lungs, pleura or mediastinum	2011	Recruitment terminated
	NCT01341496	Tumor-cell vaccines and iscomatrix with chemotherapy after tumor removal	2011	Recruitment terminated
	NCT00197912	Dendritic-cell-based therapy of malignant melanoma	2005	Completed
	NCT00093678	Celecoxib in managing pain, weight loss and weakness in patients with advanced cancer	2004	Withdrawn
	NCT02839694	Adjuvant oral decitabine and tetrahydrouridine, with or without celecoxib, in people undergoing pulmonary metastasectomy	2016	Withdrawn
	NCT02054104	Adjuvant tumor lysate vaccine and iscomatrix with or without metronomic oral cyclophosphamide and celecoxib in patients with malignancies involving lungs, esophagus, pleura or mediastinum	2014	Suspended
